# Supplementary material for: IL-2 immunotherapy rescues irradiation-induced T cell exhaustion in mouse colon cancer
Source: iScience. 2025 May 13;28(6):112639. doi: 10.1016/j.isci.2025.112639 (PMC12158513; doi:10.1016/j.isci.2025.112639)
Supplement: Document S1. Figures S1–S3 and Table S1 [file mmc1.pdf]

iScience, Volume *28*

Supplemental information

IL-2 immunotherapy rescues irradiation-induced T cell exhaustion in mouse colon cancer

Carmen S.M. Yong, Irma Telarovic, Lisa Gregor, Miro E. Raeber, Martin Pruschy, and Onur Boyman

Figure S1

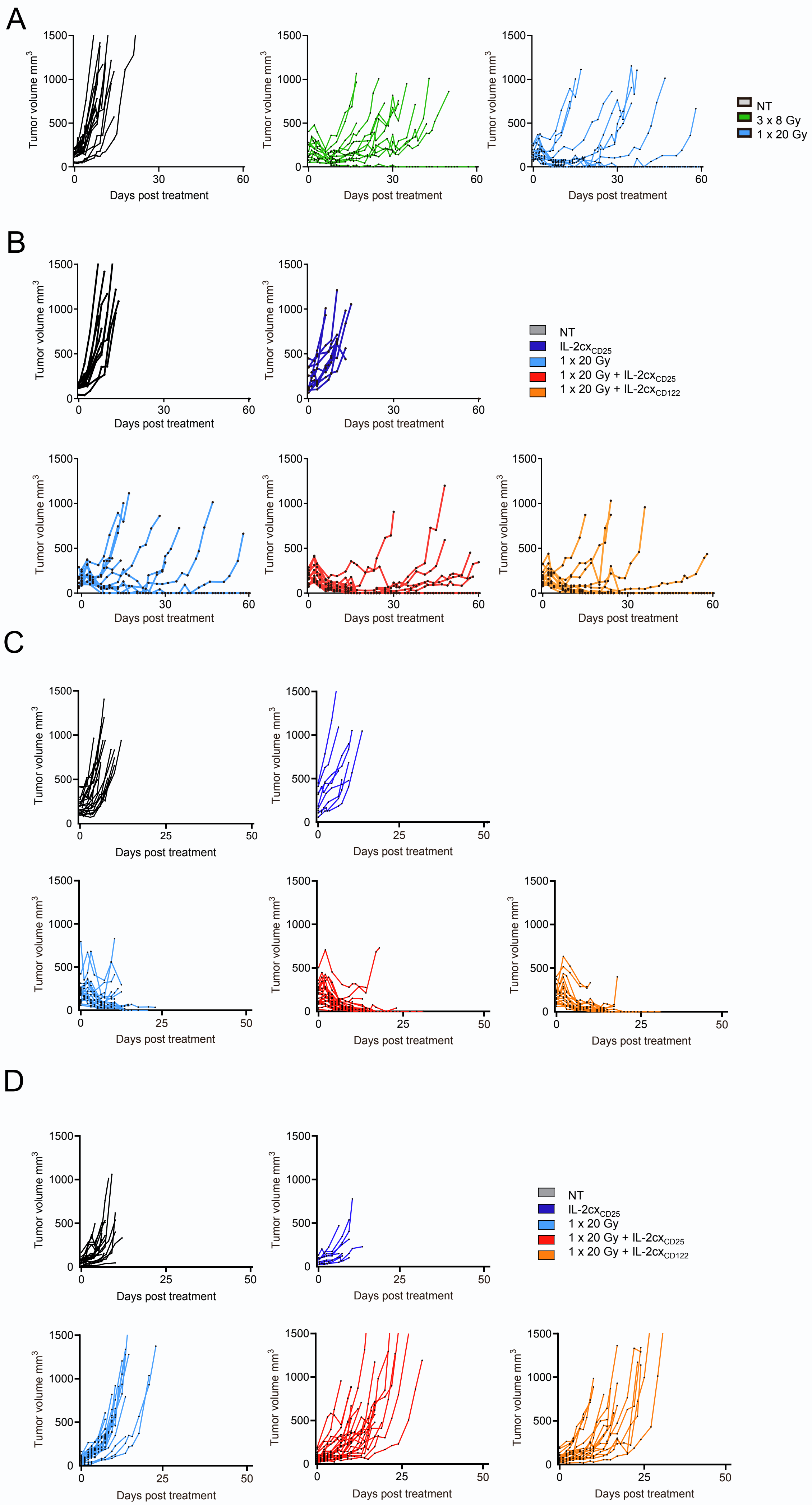
A
B
C
D
Tumor volume mm3
Days post treatment
0
500
1000
1500
30
60
25
50
NT
3 x 8 Gy
1 x 20 Gy
IL-2cxCD25
1 x 20 Gy + IL-2cxCD25
1 x 20 Gy + IL-2cxCD122

Figure S1. Individual tumor growth curves, related to Figure 1, Figure 2 and Figure 6. C57BL/6 mice were injected subcutaneously with MC38 cells, and tumors were irradiated 10–11 days later. (**A**) Mice were treated with 8 Gy on three consecutive days or a single dose of 20 Gy. (**B**) Mice were treated with a single dose of 20 Gy followed by treatment with IL-2 complexes (IL-2cx) 48 hours after irradiation for three consecutive days. (**C** and **D**) C57BL/6 mice were injected subcutaneously with MC38 cells on the right flank and abscopal tumors were inoculated in the same fashion 3 days later on the left flank of the mouse. Primary tumors were irradiated 10–11 days later with a single dose of 20 Gy, followed by treatment with IL-2 complexes 48 hours after irradiation for three consecutive days. Tumor growth curves are shown until day 60 after treatment. Data are represented as mean ± SEM of two to five independent experiments. For n values, refer to the main Figures. IL-2cx$_{CD25}$, CD25-biased IL-2/UFKAcx; IL-2cx$_{CD122}$, CD122-biased IL-2/NARAcx; NT, non-treated.

Figure S2

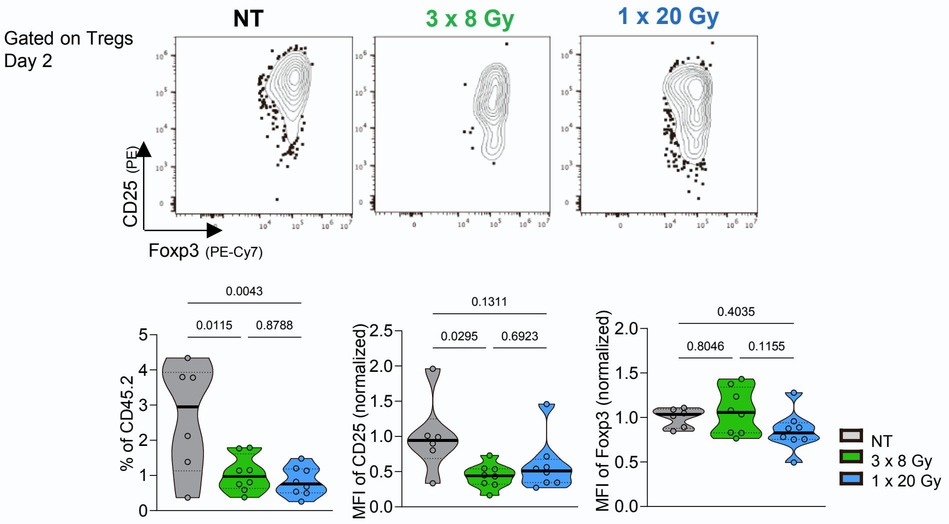
NT
3 x 8 Gy
1 x 20 Gy
Gated on Tregs
Day 2
CD25 (PE)
Foxp3 (PE-Cy7)
0.0043
0.0115
0.8788
% of CD45.2
0.1311
0.0295
0.6923
MFI of CD25 (normalized)
0.4035
0.8046
0.1155
MFI of Foxp3 (normalized)
NT
3 x 8 Gy
1 x 20 Gy

Figure S2. Irradiation reduces CD25 and Foxp3 expression in tumor-infiltrating regulatory T cells, related to Figure 1. C57BL/6 mice were injected subcutaneously with MC38 cells and tumors were irradiated 10–11 days later. Mice were treated with 8 Gy on three consecutive days or a single dose of 20 Gy. (Top) Representative flow cytometry plots gated on tumor-infiltrating $CD4^{+}Foxp3^{+}$ regulatory T cells (Tregs) on day 2 post irradiation. (Bottom) Percentage of Tregs within tumor-infiltrating $CD45.2^{+}$ immune cells and mean fluorescence intensity (MFI, normalized to non-treated mice) values of CD25 and Foxp3 expression on tumor-infiltrating Tregs. Data are represented as mean ± SEM of two to three independent experiments. n values for NT = 6, 3 x 8 Gy = 8, 1 x 20 Gy = 8. Differences were analyzed using a one-way ANOVA.

Figure S3

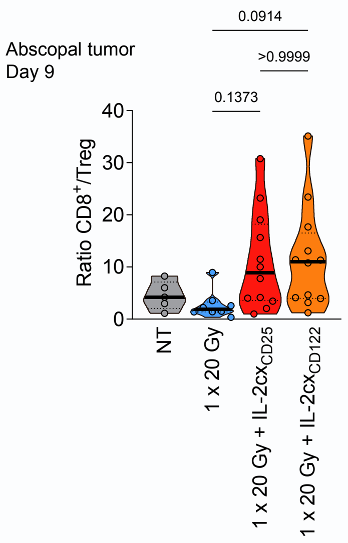

Abscopal tumor
Day 9
0.0914
>0.9999
0.1373
Ratio CD8+/Treg
40
30
20
10
0
NT
1 x 20 Gy
1 x 20 Gy + IL-2cxCD25
1 x 20 Gy + IL-2cxCD122

Figure S3. A favorable CD8-to-Treg ratio is observed in non-irradiated abscopal tumors after combination therapy, related to Figure 6. C57BL/6 mice were injected subcutaneously with MC38 cells. 10–11 days after tumor inoculation, mice were irradiated with 20 Gy and, 48 hours after irradiation, mice received indicated IL-2cx for three consecutive days. Mice were sacrificed nine days after irradiation and tumors were analyzed for percentages of $CD8^+$ T cells, $CD4^+$ T cells and $Foxp3^+$ Tregs. Ratios of $CD8^+$ T cells to Tregs in non-irradiated abscopal tumors of indicated animals are depicted. Data are represented as mean ± SEM of three independent experiments. n values for NT = 5, 1 x 20 Gy = 10, 1 x 20 Gy + IL-$2cx_{CD25/}$ IL-$2cx_{CD122}$ = 13. Differences were analyzed using a one-way ANOVA.

Table S1. Antibodies used for flow cytometry.

**Antigen**	**Fluorochrome**	**Clone**	**Manufacturer**	**Dilution**
CD122	APC	TM-β1	BioLegend	1:300
CD25	PE	PC61	BioLegend	1:300
CD39	PerCP-eFluor710	24DMS1	Invitrogen	1:300
CD4	BUV496	GK1.5	BD	1:400
CD44	BV510	IM7	BD	1:400
CD45.2	Alexa Fluor 700	104	BioLegend	1:250
CD8	APC	53-6.7	BioLegend	1:400
Fixable viability dye	eFluor780		eBioscience	1:1000
Foxp3	PE-Cy7	FJK-16s	Invitrogen	1:200
Ki67	BV605	16A8	BioLegend	1:200
NK1.1	BV711	PK136	BioLegend	1:400
PD-1	BV605	29F.1A12	BioLegend	1:300
TCRβ	BUV563	H57-597	BD	1:400
TOX	PE	TXRX10	Invitrogen	1:200
